# SIRT7 activates p53 by enhancing PCAF-mediated MDM2 degradation to arrest the cell cycle

**DOI:** 10.1038/s41388-020-1305-5

**Published:** 2020-05-13

**Authors:** Ya-Fei Lu, Xiao-Peng Xu, Xiao-Peng Lu, Qian Zhu, Ge Liu, Yan-Tao Bao, He Wen, Ying-Lu Li, Wei Gu, Wei-Guo Zhu

**Affiliations:** 1 Guangdong Key Laboratory of Genome Instability and Human Disease, Shenzhen University International Cancer Center, Department of Biochemistry and Molecular Biology, Shenzhen University School of Medicine, Shenzhen, 518055 China; 2 Institute for Cancer Genetics, College of Physicians and Surgeons, Columbia University, New York, NY 10032 USA; 3 Key Laboratory of Carcinogenesis and Translational Research (Ministry of Education), Beijing Key Laboratory of Protein Posttranslational Modifications and Cell Function, Department of Biochemistry and Molecular Biology, School of Basic Medical Sciences, Peking University Health Science Center, Beijing, 100191 China; 4 Peking University—Tsinghua University Center for Life Sciences, Beijing, 100871 China

**Keywords:** Cancer therapeutic resistance, Acetylation

## Abstract

Sirtuin 7 (SIRT7), an NAD^+^-dependent deacetylase, plays vital roles in energy sensing, but the underlying mechanisms of action remain less clear. Here, we report that SIRT7 is required for p53-dependent cell-cycle arrest during glucose deprivation. We show that SIRT7 directly interacts with p300/CBP-associated factor (PCAF) and the affinity for this interaction increases during glucose deprivation. Upon binding, SIRT7 deacetylates PCAF at lysine 720 (K720), which augments PCAF binding to murine double minute (MDM2), the p53 E3 ubiquitin ligase, leading to accelerated MDM2 degradation. This effect results in upregulated expression of the cell-cycle inhibitor, p21^Waf1/Cip1^, which further leads to cell-cycle arrest and decreased cell viability. These data highlight the importance of the SIRT7–PCAF interaction in regulating p53 activity and cell-cycle progression during conditions of glucose deprivation. This axis may represent a new avenue to design effective therapeutics based on tumor starvation.

## Introduction

Sirtuin 7 (SIRT7) is a nicotinamide adenine dinucleotide (NAD^+^)-dependent histone deacetylase that predominantly localizes to the nucleus. It has been implicated in diverse cellular processes, including aging, DNA repair, tumorigenesis, and metabolism [1–4]. SIRT7 is activated by an elevated NAD^+^/NADH ratio [5], thus SIRT7 has emerged as an important energy sensor in mammals [6, 7]. For example, SIRT7 redistributes from the nucleolus to the nucleoplasm during glucose deprivation, permitting hyperacetylation of polymerase-associated factor 53 (PAF53), which inhibits rDNA transcription to preserve energy [8]. In addition, SIRT7 arginine methylation helps coordinate glucose availability with mitochondria biogenesis to maintain energy balance [9]. SIRT7 deacetylates phosphoglycerate kinase 1 (PGK1), resulting in reduced glycolysis and liver cancer cell proliferation [10]. Moreover, we previously reported that SIRT7 promotes gluconeogenesis by modulating glucose-6-phosphatase (G6PC) expression to maintain blood glucose homeostasis upon glucose starvation [11]. While these studies support the importance of SIRT7 in glucose sensing and maintaining metabolic balance, the regulatory mechanisms require further investigation.

Cell-cycle progression is an energetically demanding process [12]. Consequently, glucose limitation can induce a reversible G1-phase arrest [13]. Given the key roles of SIRT7 in energy sensing and signaling, it is possible that SIRT7 involves in the regulation of cell cycle during glucose starvation. The tumor suppressor p53 is a master regulator of DNA replication and cell division [14], has also an essential role in glucose starvation-triggered cell-growth arrest [13]. The cyclin-dependent kinase inhibitor 1 (p21^Waf1/Cip1^) is a major target of p53 activity [15], and couples glucose starvation to cell-cycle arrest. Murine double minute (MDM2), COP1, and Pirh2 are p53-specific E3 ligases that negatively regulate p53 turnover and activity [16–20]. Under low-glucose conditions, activated AMPK phosphorylates p53 to increase p53’s ability to recruit CBP/p300, then activates cell-growth arrest [13, 21, 22]. PPAR gamma coactivators-1 (PGC-1α) also determines p53-mediated cell fate during glucose starvation [23]. These studies demonstrate the indispensable roles of p53 in regulating cell-cycle arrest and metabolic homeostasis. Therefore, it is intriguing to investigate whether SIRT7 involves in regulating p53 turnover and activity in response to glucose restriction.

p300/CBP-associated factor (PCAF) is a GCN5-related N-acetyltransferase that localizes to the nucleus [4] and regulates many cellular processes, including transcription [24], proliferation [10], differentiation [25, 26], apoptosis [27, 28], and DNA damage repair [29]. PCAF is acetylated/deacetylated to regulate its histone acetyltransferase (HAT) activity and cellular translocation [30–32]. Interestingly, PCAF also possesses E3 ubiquitin ligase activity for MDM2 and can stabilize p53 protein levels [33]. Therefore, understanding how PCAF ubiquitination activity is regulated is critical for targeting p53 activity. Recent studies have also identified that PCAF has broader roles in cell metabolism. For example, PCAF regulates glycolysis by acetylating glyceraldehyde-3-phosphate dehydrogenase or PGK1 enhance glycolysis and cell proliferation [10, 34]. PCAF also promotes de novo lipid synthesis by acetylating ATP-citrate lyase [35]. In addition, PCAF has critical roles in improving glucose homeostasis and insulin sensitivity in diet-induced obese and ob/ob mouse models by regulating PGC-1α acetylation and proteasomal degradation [36]. It thus is clear that PCAF functions as an important regulator in maintaining metabolism balance.

In this study, we confirm the important roles for SIRT7 in regulating p53-dependent cell-cycle arrest upon glucose deprivation. SIRT7 directly interacts with and deacetylates PCAF, which promotes PCAF binding to MDM2 and consequential degrades MDM2 to enhance p53 stability. Both SIRT7 depletion and a PCAF acetylation-mimic mutation block cell-cycle arrest and result in increased cell viability during glucose deprivation. Overall, SIRT7-dependent PCAF deacetylation is critical for arresting cell proliferation and inhibiting cell survival upon glucose depletion.

## Results

### SIRT7 is required for p53-dependent cell-cycle arrest upon glucose deprivation

**Fig. 1:**
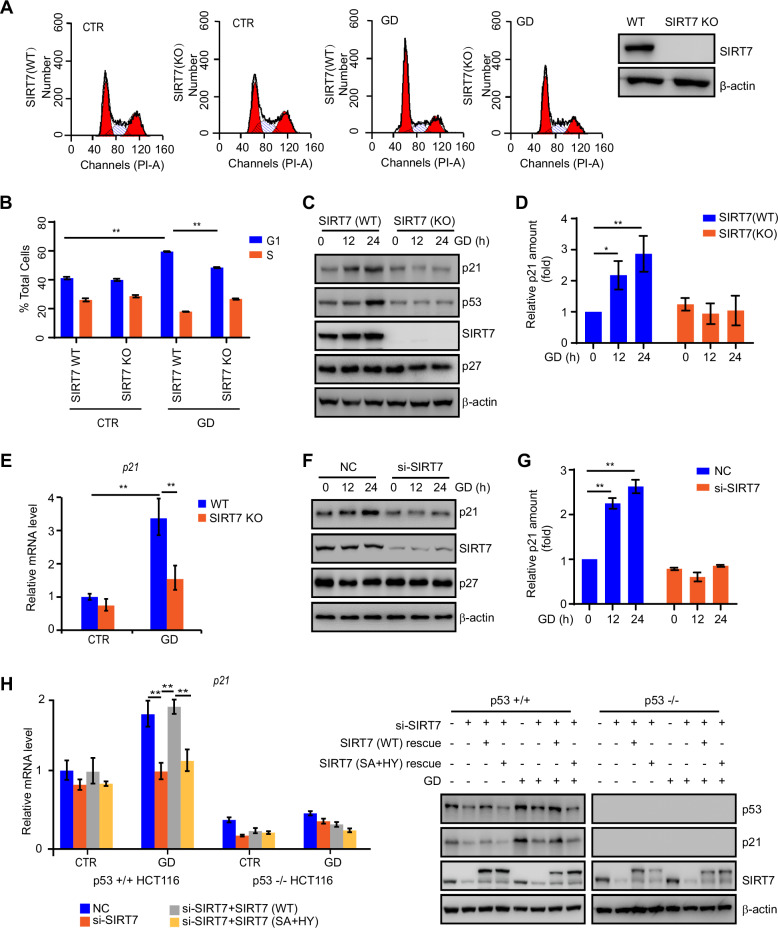
SIRT7 is required for p53-dependent cell-cycle arrest upon glucose deprivation. **a** SIRT7 (WT) and SIRT7 (KO) HCT116 cells were analyzed by immunoblotting (right panel). SIRT7 (WT) and SIRT7 (KO) HCT116 cells were cultured in normal medium (CTR) or subjected to glucose deprivation (GD) for 12 h. Cells were harvested, stained with propidium iodide (PI), and then analyzed by FACS. **b** The bars represent the percentage cells of indicated cell-cycle stage. The data represent the means ± SD (*n* = 3 experiments), unpaired two-tailed Student’s *t* test, **p < 0.01. **c** SIRT7 (WT) and SIRT7 (KO) HCT116 cells were subjected to glucose deprivation (GD) for the indicated times and whole cell lysates were analyzed by immunoblotting with the indicated antibodies. β-actin was used as a loading control. **d** Quantitation of p21 expression levels. Immunoblots in **c** were scanned and normalized to β-actin. The data represent the means ± SD (*n* = 3–4 experiments), One-way ANOVA, *p < 0.05, **p < 0.01, compared with the indicated group. **e** SIRT7 (WT) and SIRT7 (KO) HCT116 cells were cultured in normal medium (CTR) or subjected to glucose deprivation (GD) for 24 h. Relative *p21* expression was determined by real-time PCR. The data represent the means ± SD (*n* = 3 experiments), unpaired two-tailed Student’s *t* test, **p < 0.01, compared with the indicated group. **f** HCT116 cells were transfected with siRNA against SIRT7 (si-SIRT7) or a negative control siRNA (NC) and subjected to glucose deprivation (GD) for the indicated times. Whole cell lysates were analyzed by immunoblotting. β-actin was used as a loading control. **g** Quantitation of p21 expression levels. Immunoblots in **f** were scanned and normalized to β-actin. The data represent the means ± SD (*n* = 3–4 experiments), One-way ANOVA, **p < 0.01, compared with the indicated group. **h** p53^+/+^ HCT116 and p53^−/−^ HCT116 cells were transfected with the indicated siRNA and plasmid combinations and then subjected to glucose deprivation (GD) for 12 h. Whole cell lysates were analyzed by immunoblotting (right panel). Relative *p21* levels were determined by real-time PCR (left pane). The data represent the means ± SD (*n* = 3 experiments), unpaired two-tailed Student’s *t* test, **p < 0.01, compared with the indicated group.

SIRT7 has been proposed as an energy sensor [7], we hypothesized that SIRT7 might be involved in regulating cell-cycle arrest under energy limitation, such as glucose deprivation. To assess the role of SIRT7 in this response, we generated SIRT7 knockout (KO) HCT116 cells using (CRISPR)-Cas9 technology (Fig. 1a), subjected them to glucose deprivation and then performed cell-cycle analyses. Unlike SIRT7 wild-type (WT) cells, the SIRT7 (KO) cells were unable to efficiently arrest the cell cycle in G1 phase during glucose deprivation (Fig. 1a, b).

Cell-cycle arrest in G1 phase is often mediated by p21 [15]. As expected, p21 protein and mRNA levels were rapidly accumulated in SIRT7 (WT), but not SIRT7 (KO) HCT116 cells after glucose deprivation (Fig. 1c–e). Elevated p21 expression upon glucose deprivation was also suppressed in SIRT7 knockdown by siRNA HCT116 and U2OS cells (Fig. 1f, g and Fig. S1A). As expected, glucose starvation-induced p21 upregulation was dependent on p53, as these changes were not remarkable in H1299 cells or p53^−/−^ HCT116 cells that lack p53 (Fig. S1B–D).

To confirm whether SIRT7 regulates p21 expression in a p53-dependent manner, we knocked down SIRT7 again in p53^+/+^ and p53^−/−^ HCT116 cells, and then monitored p21 expression. We found impaired p53-dependent p21 expression upon glucose deprivation in si-SIRT7 p53^+/+^ HCT116 cells, which was restored upon reintroduction of SIRT7 (WT) but not catalytically dead mutant SIRT7 (S111A/H187Y). However, these changes were almost abolished in p53^−/−^ HCT116 cells (Fig. 1h), suggesting SIRT7-mediated cell-cycle arrest is dependent on p53. Together, these results indicate that SIRT7 is required for p53-mediated cell-cycle arrest upon glucose deprivation.

### SIRT7 regulates p53 stability

**Fig. 2:**
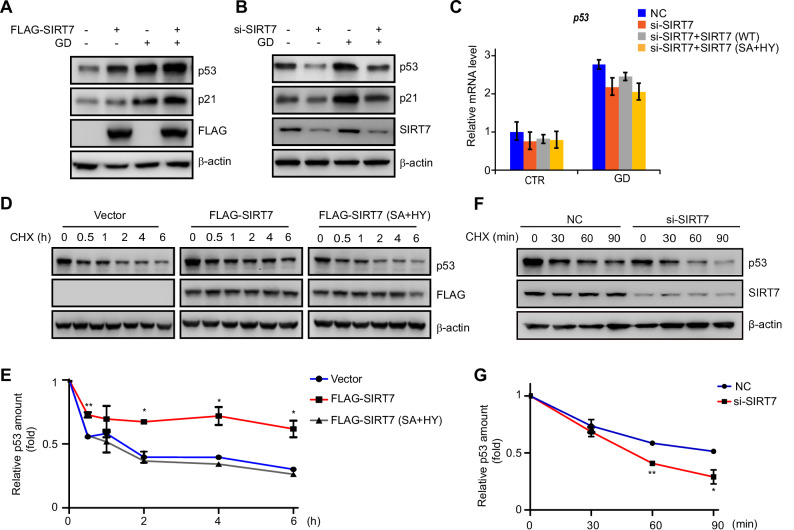
SIRT7 regulates p53 stability. HCT116 cells were transfected with FLAG-SIRT7 (**a**) or SIRT7 siRNA (**b**) and subjected or not to glucose starvation (GD) for 12 h. Whole cell lysates were analyzed by immunoblotting. **c** HCT116 cells were transfected with the indicated siRNAs or plasmids, and then subjected or not to glucose deprivation (GD) for 12 h. Relative *p53* expression levels were determined by real-time PCR. The data represent the means ± SD (*n* = 3 experiments), unpaired two-tailed Student’s *t* test, no significance *p* > 0.05. **d** HCT116 cells were transfected with the indicated plasmids and then treated with cycloheximide (CHX) (30 μg/mL) for the indicated times. Whole cell lysates were analyzed by immunoblotting. **e** Quantitation of p53 protein levels. Immunoblots in **d** were scanned and normalized to β-actin. **f** HCT116 cells were transfected with the indicated siRNAs and then treated with cycloheximide CHX (30 μg/mL) for the indicated times. Whole cell lysates were analyzed by immunoblotting. β-actin was used as a loading control throughout. **g** Quantitation of p53 protein levels. Immunoblots in **f** were scanned and normalized to β-actin. The data (**e**, **g**) represent the means ± SD (*n* = 3–4 experiments), unpaired two-tailed Student’s *t* test, *p < 0.05, **p < 0.01, compared with the indicated time of vector or NC group.

Next, we explored the connection between SIRT7 and p53. We found that the activated p53 expression during glucose starvation was increased in FLAG-SIRT7 overexpressing HCT116 cells (Fig. 2a), but suppressed in SIRT7 knockdown HCT116 and U2OS cells (Fig. 2b and Fig. S2A). Interestingly, *p53* expression levels remained unaffected (Fig. 2c), indicating that SIRT7 may regulate p53 protein stability. We thus separately transfected HCT116 cells with SIRT7 (WT) and enzyme activity dead SIRT7 (SA/HY), and then treated with cycloheximide (CHX), a protein synthesis inhibitor. As shown in Fig. 2d, e, SIRT7 (WT) increased the half-life of endogenous p53, whereas SIRT7 (SA/HY) had no effect. Overexpression of SIRT7 (WT) also led to increased p53 stability in U2OS cells (Fig. S2B). Conversely, knockdown SIRT7 by siRNA in HCT116 or U2OS cells led to a reversed result (Fig. 2f, g and Fig. S2C). We also examined the ability of SIRT7 to deacetylate p53. K382/373-acetylated p53 remained virtually unchanged in SIRT7 knockdown HCT116 using siRNA after treatment with MG132, a proteasome inhibitor (Fig. S2D), our results are consistent with the previous report that SIRT7 does not deacetylate p53 in vitro or in HT1080 or NHF cells [37, 38]. These data first demonstrate that the SIRT7-mediated increase in p53 expression is achieved by regulating p53 stability.

### SIRT7 promotes MDM2 degradation

The data led us to propose that SIRT7 may regulate the E3 ligase(s) involved in p53 degradation. To assess this proposal, we again knocked down SIRT7 by siRNA in U2OS cells and then treated with CHX before assessing the half-life of the p53’s primary E3 ligases: Pirh2 [19] and COP1 [18]. Here, knockdown of SIRT7 had no effect on Pirh2 or COP1 stability (Fig. S3A). Interestingly, SIRT7 knockdown increased endogenous MDM2 protein levels (Fig. S3B), whereas overexpression of FLAG-SIRT7 decreased endogenous MDM2 protein levels (Fig. S3C).

**Fig. 3:**
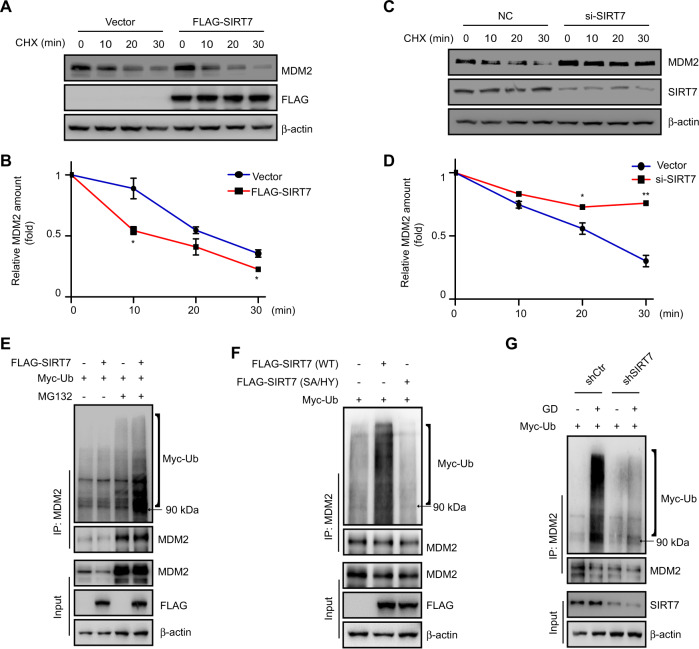
SIRT7 promotes MDM2 degradation. **a** HCT116 cells were transfected with the indicated plasmids and then treated with cycloheximide (CHX) (30 μg/mL) for the indicated times. Whole cell lysates were analyzed by immunoblotting. **b** Quantitation of MDM2 protein levels. Immunoblots in **a** were scanned and normalized to β-actin. **c** HCT116 cells were transfected with the indicated siRNAs and then treated with CHX (30 μg/mL) for the indicated times. Whole cell lysates were analyzed by immunoblotting. β-actin was used as a loading control throughout. **d** Quantitation of MDM2 protein levels. Immunoblots in **c** were scanned and normalized to β-actin. The data (**b**, **d**) represent the means ± SD (*n* = 3 experiments), unpaired two-tailed Student’s *t* test, *p < 0.05, **p < 0.01, compared with the indicated time of vector or NC group. **e** HCT116 cells were transfected with the indicated plasmids and treated or not with 10 μM MG132 for 8 h. Whole cell lysates were prepared and subjected to immunoprecipitation with an anti-MDM2 antibody before immunoblotting. **f** HCT116 cells were transfected with the indicated plasmids and treated with 10 μM MG132 for 8 h. Cell lysates were prepared and subjected to immunoprecipitation with an anti-MDM2 antibody before immunoblotting. **g** Stable SIRT7 knockdown (shSIRT7) or control (shCtr) HCT116 cells were subjected to 10 μM MG132 treated with or without glucose deprivation (GD) for 12 h. Cell lysates were prepared and subjected to immunoprecipitation with an anti-MDM2 antibody before immunoblotting.

Next, we sought to determine whether SIRT7 regulates MDM2 stability. We transfected FLAG-SIRT7 into HCT116 cells and then treated with CHX. As shown in Fig. 3a, b, the half-life of endogenous MDM2 was dramatically decreased. Conversely, knockdown of SIRT7 by siRNA resulted in a reversed result (Fig. 3c, d). As MDM2 is degraded by the ubiquitin–proteasome pathway [39], we next investigated whether SIRT7 affects MDM2 ubiquitination levels. We cotransfected HCT116 cells with FLAG-SIRT7 and Myc-Ub, and then monitored endogenous MDM2 ubiquitination levels. We detected elevated polyubiquitinated MDM2 in the cotransfected cells after treatment with MG132, compared with control cells transfected with Myc-Ub alone (Fig. 3e). However, transfection of an enzymatic-dead SIRT7 mutant (SA/HY) did not increase MDM2 polyubiquitination levels in both HCT116 and U2OS cells (Fig. 3f and Fig. S3D), indicating that SIRT7 deacetylase activity is also required to modulate MDM2 polyubiquitination.

Myc-Ub transfection is used to add sensitivity to the MDM2 ubiquitination analysis. The polyubiquitination levels of endogenous MDM2 were increased in SIRT7 overexpression HCT116 cells without Myc-Ub (Fig. S3E). In addition, we found that MDM2 polyubiquitination levels were increased upon glucose deprivation, whereas this effect was largely abrogated in SIRT7 stable knockdown (shSIRT7) HCT116 cells (Fig. 3g). Moreover, glucose deprivation-induced MDM2 degradation was mainly absent in shSIRT7 U2OS cells (Fig. S3F). These data suggest that glucose deprivation-induced MDM2 degradation is dependent on SIRT7. Consistent with a previous observation [40], SIRT7 could interact with MDM2, whereas the affinity for this interaction remained largely unaltered after glucose deprivation (Fig. S3G). In addition, SIRT7 did not deacetylate MDM2 (Fig. S3H), suggesting that SIRT7 may indirectly regulate MDM2 stability.

### Glucose deprivation enhances the interaction between SIRT7 and PCAF

**Fig. 4:**
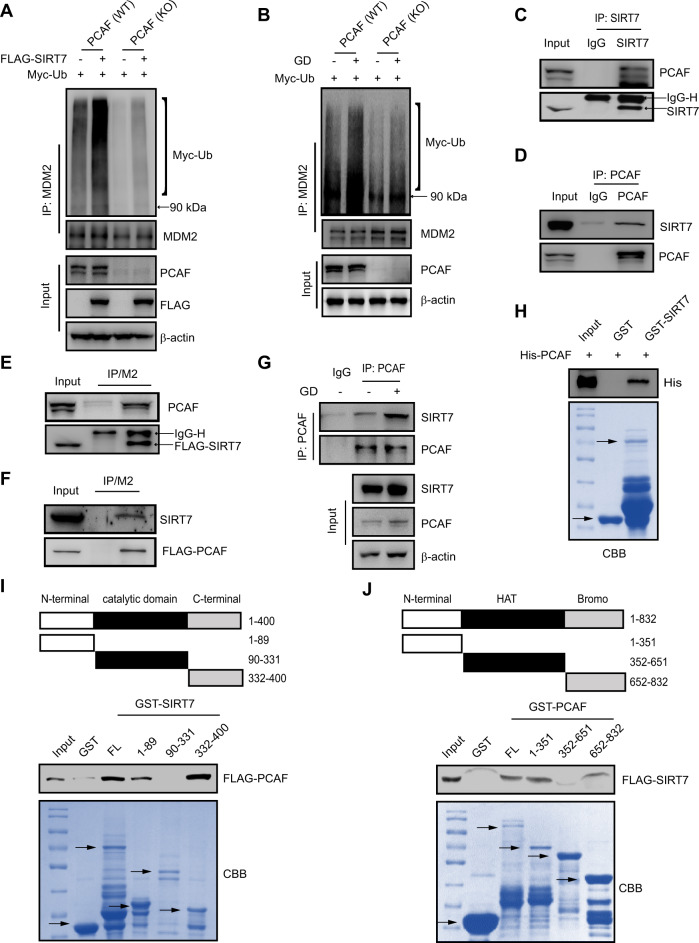
Glucose deprivation (GD) enhances the interaction between SIRT7 and PCAF. **a** PCAF (WT) and PCAF knockout (KO) cells were transfected with the indicated plasmids and treated with 10 μM MG132 for 8 h. Cell lysates were prepared and immunoprecipitated with anti-MDM2 antibodies before immunoblotting. **b** PCAF (WT) and PCAF (KO) HCT116 cells were subjected to glucose deprivation (GD) and 10 μM MG132 treated for 12 h. Cell lysates were prepared and subjected to immunoprecipitation with an anti-MDM2 antibody before immunoblotting. **c**, **d** HCT116 cells lysates were immunoprecipitated using an anti-IgG, anti-SIRT7 antibody, or anti-PCAF antibody and analyzed by immunoblotting. **e**, **f** HCT116 cells were transfected with FLAG-SIRT7, FLAG-PCAF, or an empty vector. Cell lysates were prepared and immunoprecipitated with FLAG-conjugated M2 beads before immunoblotting. **g** HCT116 cells were transfected with the indicated plasmids and subjected to glucose deprivation (GD) for 12 h. Cell lysates were prepared and immunoprecipitated with anti-PCAF antibody before immunoblotting. **h** Full-length His-PCAF fusion protein was incubated with GST or GST-SIRT7. Protein-bound glutathione-agarose beads were analyzed by immunoblotting and the corresponding gels were stained with Coomassie brilliant blue (CBB). The arrows indicate proteins with correct molecular masses (bottom panel). **i** Schematic of SIRT7 and domain constructs fused with GST (top panel). HCT116 cells were transfected with FLAG-PCAF. Cell lysates were prepared and incubated with GST, full-length (FL) SIRT7, or one of three GST-fusion SIRT7 domains. Protein-bound glutathione-agarose beads were analyzed by immunoblotting and the corresponding gels were stained with CBB. The arrows indicate proteins with correct molecular masses (bottom panel). **j** A schematic PCAF and domain constructs fused with GST (top panel). HCT116 cells were transfected with FLAG-SIRT7. Cell lysates were prepared and incubated with GST, FL PCAF, or one of three GST-fusion PCAF domains. Protein-bound glutathione-agarose beads were analyzed by immunoblotting and the corresponding gels were stained with CBB. The arrows indicate proteins with correct molecular masses (bottom panel).

Because PCAF possesses ubiquitination activity to control MDM2 turnover [33], we thus examined whether SIRT7-regulated MDM2 degradation is dependent on PCAF. PCAF (KO) HCT116 cells were generated using (CRISPR)-Cas9 technology. We found no remarkable difference in MDM2 polyubiquitination levels or stability between PCAF (KO) cells overexpressing FLAG-SIRT7 and PCAF (KO) cells overexpressing an empty vector (Fig. 4a and Fig. S4A). In addition, MDM2 polyubiquitination levels were markedly increased during glucose deprivation, whereas this effect was absent in PCAF (KO) cells (Fig. 4b). These results indicate that SIRT7 may regulate MDM2 polyubiquitination in a PCAF-dependent manner.

Subsequently, we determined the protein interaction between SIRT7 and PCAF by co-immunoprecipitation (Co-IP) assay using SIRT7 and PCAF antibodies. We detected a clear interaction between SIRT7 and PCAF in HCT116 (Fig. 4c, d) and U2OS cells (Fig. S4B, C). In addition, we detected an interaction between PCAF and exogenously expressed FLAG-SIRT7 and reciprocally SIRT7 with exogenously expressed FLAG-PCAF in HCT116 cells (Fig. 4e, f). Moreover, this interaction between SIRT7 and PCAF was elevated upon glucose starvation in HCT116 cells (Fig. 4g) and U2OS cells (Fig. S4D).

To further investigate whether SIRT7 directly interacts with PCAF, an in vitro glutathione S-transferase (GST) pull-down assay was performed. Here, we purified the full-length GST-tagged SIRT7 protein, and incubated it with purified His-PCAF protein under properly conditions. We found that PCAF bound to GST-SIRT7, suggesting that SIRT7 directly interacts with PCAF (Fig. 4h). To map the binding regions between PCAF and SIRT7, we performed GST pull-down experiments again. We constructed and expressed three GST-SIRT7 fragments according to the SIRT7 functional domains: the N terminus (1–89 aa), the deacetylase core domain (90–331 aa), and the C terminus (332–400 aa). GST pull-down assays using HCT116 cell lysates containing FLAG-PCAF showed that the SIRT7 N terminus and C terminus interacted with PCAF (Fig. 4i). We similarly generated three GST-PCAF fragments based on the N terminus (1–351 aa), the HAT domain (352–651 aa) and the bromo domain (652–833 aa). Here, SIRT7 mainly bound to the PCAF N terminus and C terminus (Fig. 4j). Collectively, these findings demonstrate that PCAF is a novel SIRT7 substrate and the two proteins show an enhanced interaction upon glucose deprivation.

### SIRT7 deacetylates PCAF at lysine 720

**Fig. 5:**
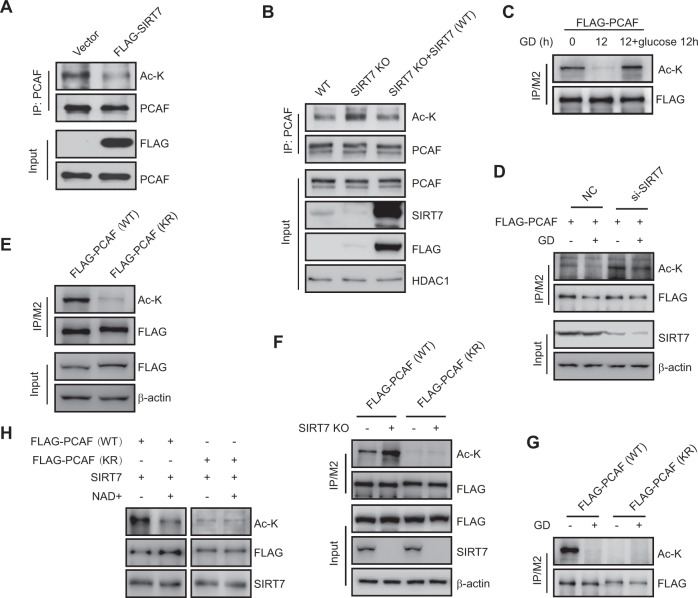
SIRT7 deacetylates PCAF at lysine 720. **a** HCT116 cells were transfected with FLAG-SIRT7 or an empty vector. Cell extracts were immunoprecipitated with an anti-PCAF antibody before immunoblotting. **b** SIRT7 (WT) or SIRT7 (KO) HCT116 cells were transfected with FLAG-SIRT7 or an empty vector. Cell extracts were immunoprecipitated with an anti-PCAF antibody before immunoblotting. **c** HCT116 cells were transfected with FLAG-PCAF and subjected to glucose deprivation (GD) for 0 or 12 h, or recultured in replete growth medium for 12 h after starvation. Cell extracts were immunoprecipitated with FLAG-conjugated M2 beads before immunoblotting. **d** HCT116 cells were transfected with FLAG-PCAF and treated with SIRT7 or negative control (NC) siRNAs before being subjected to glucose deprivation (GD) for 12 h. Cell extracts were immunoprecipitated with FLAG-conjugated M2 beads before immunoblotting. **e** HCT116 cells were transfected with WT or K720R mutant FLAG-PCAF plasmids. Cell extracts were immunoprecipitated with FLAG-conjugated M2 beads before immunoblotting. **f** SIRT7 (WT) or SIRT7 knockout (KO) cells were transfected with WT or K720R mutant FLAG-PCAF plasmids and cell lysates were immunoprecipitated with FLAG-conjugated M2 beads before immunoblotting. **g** HCT116 cells were transfected with WT or K720R mutant FLAG-PCAF plasmids and subjected to glucose deprivation (GD) for 12 h. Cell lysates were immunoprecipitated with FLAG-conjugated M2 beads before immunoblotting. **h** M2 bead-bound FLAG-PCAF (WT) or FLAG-PCAF (KR) proteins were incubated with purified FLAG-SIRT7 proteins in the presence of NAD^+^. Total acetylation levels of FLAG-PCAF were determined by immunoblotting.

To determine whether SIRT7 deacetylates PCAF, we next examined PCAF acetylation levels in FLAG-SIRT7 overexpressing HCT116 and U2OS cells. SIRT7 overexpression led to decrease in endogenous PCAF acetylation levels (Fig. 5a and Fig. S5A). To support this finding, we performed a rescue assay by transfecting SIRT7 (WT) into SIRT7 (KO) cells. We found that PCAF acetylation levels increased in SIRT7 (KO) cells compared with control cells, whereas reintroduction of SIRT7 (WT) could restore this effect (Fig. 5b), indicating that SIRT7 deacetylates PCAF in vivo. In addition, glucose deprivation markedly decreased PCAF acetylation levels in HCT116 and U2OS cells, whereas these effects were reversed upon glucose supplementation (Fig. 5c and Fig. S5B). However, the reduction in PCAF acetylation levels upon glucose deprivation were abrogated in SIRT7 knockdown cells (Fig. 5d).

We next sought to identify which PCAF lysine resides are deacetylated by SIRT7. We transfected SIRT7 (WT) or SIRT7 (KO) cells with FLAG-PCAF and then performed immunoprecipitation experiments pulling down FLAG before analyzing the acetylation levels on FLAG-PCAF lysine residues by mass spectrometry. We found an increased acetylation signal at lysine 720 site (Fig. S5D) and this site is highly conserved across multiple species (Fig. S5E). To confirm acetylation at this site, we mutated PCAF (K720) to mimic the nonacetylated form by replacing lysine with arginine (K720R) or to mimic acetylation by replacing lysine with glutamine (K720Q). We then transfected HCT116 cells with PCAF (WT) or PCAF (K720R) and then performed immunoprecipitation experiments. Here, the acetylation levels of PCAF (K720R) mutant were abolished compared with control (Fig. 5e). In addition, the acetylation levels of PCAF (WT), but not PCAF (K720R), were increased in SIRT7 (KO) cells compared with SIRT7 (WT) cells (Fig. 5f). Moreover, glucose deprivation-induced PCAF deacetylation was also abolished in HCT116 cells expressing PCAF (K720R) mutant (Fig. 5g).

We finally performed an in vitro deacetylation assay. M2 bead-purified PCAF (WT) and PCAF (K720R) proteins were separately incubated with purified SIRT7 protein under in vitro deacetylation conditions. We found that the PCAF (WT) acetylation levels were decreased in the presence of SIRT7 and NAD^+^, whereas PCAF (K720R) acetylation levels remained unchanged (Fig. 5h). Together, these data implicate that SIRT7 deacetylates PCAF at K720.

### SIRT7 augments PCAF binding to MDM2

**Fig. 6:**
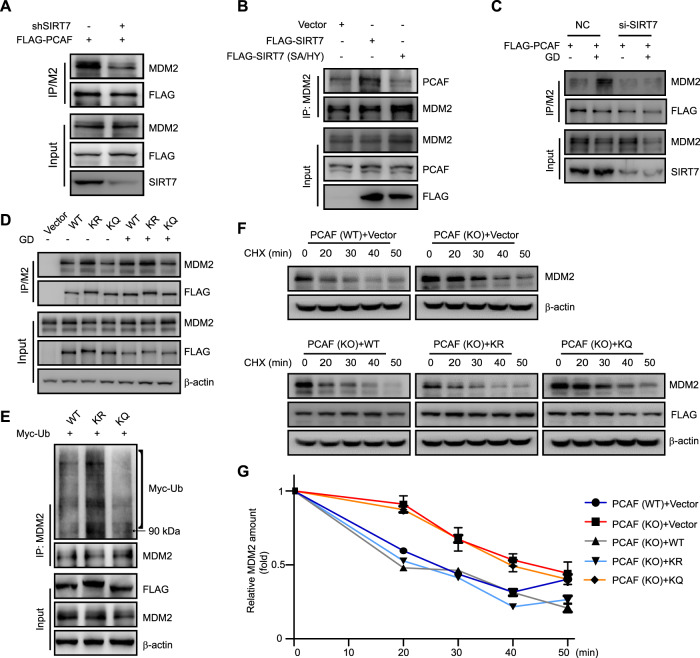
SIRT7 augments PCAF binding to MDM2. **a** Stable SIRT7 knockdown (shSIRT7) or control (shCtr) HCT116 cells were transfected with FLAG-PCAF and treated with 10 μM MG132 for 8 h. Cell lysates were subjected to immunoprecipitation with FLAG-conjugated M2 beads before immunoblotting. **b** HCT116 cells were transfected with the indicated SIRT7 plasmids or an empty vector and treated with 10 μM MG132 for 8 h. Cell lysates were subjected to immunoprecipitation with an anti-MDM2 antibody before immunoblotting. **c** HCT116 cells were transfected with SIRT7 (si-SIRT7) or negative control (NC) siRNAs and transfected with FLAG-PCAF before being subjected to glucose deprivation (GD) and 10 μM MG132 treated for 12 h. Total cell lysates were immunoprecipitated with FLAG-conjugated M2 beads before immunoblotting. **d** HCT116 cells were transfected with the indicated FLAG-PCAF plasmids or an empty vector and treated with 10 μM MG132 for 8 h before being subjected to 10 μM MG132 treated with or without glucose deprivation (GD) for 12 h. Total cell lysates were immunoprecipitated with FLAG-conjugated M2 beads before immunoblotting. **e** HCT116 cells were transfected with the indicated FLAG-PCAF and Myc-Ub plasmids and treated with 10 μM MG132 for 8 h. Total cell lysates were immunoprecipitated with an anti-MDM2 antibody and the ubiquitination levels were analyzed by immunoblotting. **f** PCAF(WT) and PCAF (KO) HCT116 cells were transfected with the indicated plasmids and then treated with CHX for the indicated time. Cells were harvested and MDM2 protein levels were measured by immunoblotting. **g** Quantitation of MDM2 protein levels. Immunoblots in **f** were scanned and normalized to β-actin. The data represent the means ± SD (*n* = 3 experiments).

Because neither knockdown nor overexpression of SIRT7 affected PCAF protein levels (Fig. S6A, B), we proposed that SIRT7 might augment the ability of PCAF to bind MDM2. We thus examined the interaction between PCAF and MDM2 in SIRT7 (WT) or shSIRT7 cells, we found that the interaction was decreased in shSIRT7 HCT116 and U2OS cells (Fig. 6a and Fig. S6C). Conversely, FLAG-SIRT7 (WT) overexpression increased the interaction between PCAF and MDM2, whereas overexpressing FLAG-SIRT7 (SA/HY) failed to do (Fig. 6b). Glucose deprivation also increased the interaction between PCAF and MDM2 (Fig. 6c and Fig. S6D), and this effect was dependent on SIRT7 (Fig. 6c). These data suggest that SIRT7 augments PCAF binding to MDM2, leading to an accelerated MDM2 degradation.

To dissect the impact of the PCAF (K720) deacetylation on PCAF–MDM2 binding, we transfected PCAF (WT), PCAF (K720R), or PCAF (K720Q) into HCT116 cells and then measured the affinity for PCAF–MDM2 binding upon glucose deprivation. We found that overexpression of PCAF (WT) and PCAF (K720R) were able to form a stronger complex with MDM2 after glucose deprivation, whereas overexpression of PCAF (K720Q) mutant had minimal effects (Fig. 6d). We also performed a Co-IP experiment to evaluate the interaction between PCAF and MDM2 in U2OS cells. As shown in Fig. S6E, MDM2 bound more strongly to PCAF (WT) or PCAF (K720R). In addition, PCAF (K720R) overexpression augmented MDM2 polyubiquitination levels compared with control (Fig. 6e). Finally, we retransfected PCAF (WT), PCAF (K720R), or PCAF (K720Q) into PCAF (KO) cells, and then treated with CHX to determine MDM2 half-life. Here, the half-life of MDM2 was increased in PCAF (KO) cells but reintroduction of PCAF (WT) or PCAF (720R) could reverse this effect (Fig. 6f, g). Together, these data indicate that PCAF K720 deacetylation increases PCAF’s ability to bind MDM2 and further accelerate MDM2 degradation.

### SIRT7-mediated PCAF deacetylation promotes cell-cycle arrest and decreases cell viability in response to glucose deprivation

**Fig. 7:**
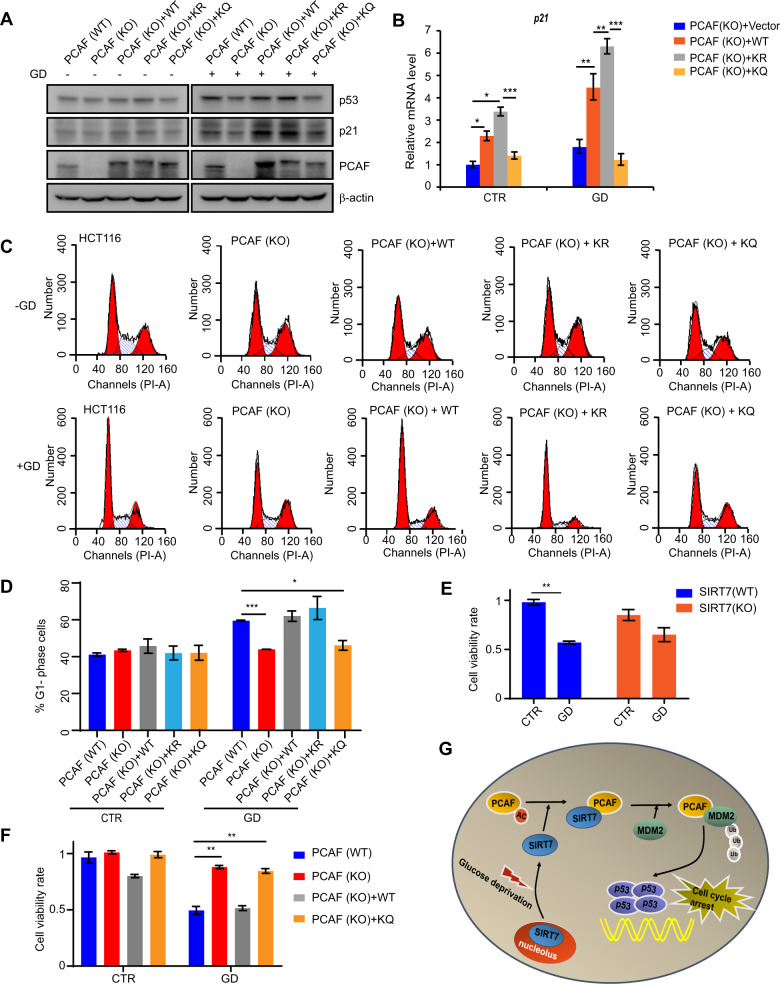
SIRT7-mediated PCAF deacetylation promotes cell-cycle arrest and decreases cell viability in response to glucose deprivation. **a** PCAF (WT) or PCAF (KO) cells were transfected with the indicated plasmids and then subjected to glucose deprivation (GD) for 12 h, whole cell lysates were analyzed by immunoblotting with the indicated antibodies. β-actin was used as a loading control. **b** PCAF (KO) cells were transfected with the indicated plasmids and then subjected to glucose deprivation (GD) for 12 h, the relative p21 mRNA levels were determined by real-time PCR. The data represent the means ± SD (*n* = 3 experiments), unpaired two-tailed Student’s *t* test, *p < 0.05, **p < 0.01, ***p < 0.001, compared with the indicated group. **c** PCAF (WT) or PCAF (KO) cells were transfected with the indicated plasmids and then subjected to glucose deprivation (+GD) or not (−GD) for 12 h. Cells were harvested and stained with propidium iodide before FACS. **d** The bars represent the percentage cells of G1 phase. The data represent the means ± SD (*n* = 3 experiments), unpaired two-tailed Student’s *t* test, *p < 0.05, ***p < 0.001. **e** SIRT7 (WT) and SIRT7 (KO) HCT116 cells were subjected to colony formation after glucose deprivation (GD) for 12 h. **f** PCAF (WT) and PCAF (KO) HCT116 cells were stable transfected with the indicated plasmids and subjected to colony formation after glucose deprivation (GD) for 12 h. The data (**e**, **f**) represent the means ± SD (*n* = 3 experiments), unpaired two-tailed Student’s *t* test, **p < 0.01. **g** Schematic model for p53 activity regulation by SIRT7-mediated PCAF deacetylation in response to glucose deprivation. Upon glucose deprivation, SIRT7 redistributes from the nucleolus to the nucleoplasm and deacetylates PCAF, leading to enhanced PCAF binding to MDM2. This enhanced binding promotes MDM2 degradation, resulting in increased p53 stability and activity to initiate cell-cycle arrest and decrease cell viability.

We next aimed to validate the role of PCAF deacetylation in glucose starvation-induced cell-cycle arrest. We performed rescue experiments by retransfecting PCAF (WT), PCAF (K720R), or PCAF (K720Q) plasmids into PCAF (KO) cells. As shown in Fig. 7a, glucose deprivation-induced increases in p53 and p21 were repressed in PCAF (KO) cells, whereas reintroduction of PCAF (WT) and PCAF (K720R) restored this effect. In addition, glucose deprivation-induced *p21* activation was upregulated in PCAF (KO) cells reintroduced with PCAF (WT) and PCAF (K720R) (Fig. 7b). Moreover, cell-cycle analysis showed that PCAF (KO) cells reintroduced with PCAF (WT) and PCAF (K720R) were able to efficiently arrest in G1 phase after glucose deprivation (Fig. 7c, d). These data indicate that SIRT7-mediated PCAF deacetylation stimulates cell-cycle arrest in G1 phase upon glucose depletion.

Finally, we sought to determine the function relevance of SIRT7 and PCAF to the cellular response to glucose deprivation. Colony formation analysis showed that SIRT7 (WT) cells were more sensitive to glucose deprivation compared with SIRT7 (KO) cells (Fig. 7e). In addition, PCAF (KO) cells were able to effectively resist to glucose depletion compared with control cells, reintroduced PCAF (WT) could reverse this effect but reintroduced PCAF (K720Q) failed to do so (Fig. 7f). Altogether, these findings indicate the roles of SIRT7-mediated PCAF deacetylation in regulating cell cycle and cell viability during glucose deprivation.

## Discussion

Our findings identify a novel mechanism for nucleoplasmic SIRT7 in the control of cell-cycle progression and cell viability during no glucose availability. Specifically, we reveal that SIRT7-mediated PCAF K720 deacetylation promotes PCAF binding to MDM2 and enhances MDM2 degradation, which accounts for increased p53 stability and activity. Overall, these effects initiate cell-cycle arrest upon glucose deprivation (Fig. 7g).

Accumulating evidence has revealed that sirtuins act as energy-sensing deacetylases and have vital roles in metabolic homeostasis mainly through substrate deacetylation [7]. SIRT7, the focus of this study, regulates gluconeogenesis by modulating G6PC expression during glucose deprivation [11]. SIRT7 (KO) mice are resistant to high-fat-diet-induced fatty liver disease and glucose intolerance [41]. SIRT7 also triggers hematopoietic stem cells to exit cell cycle and maintain quiescence [42]. Among its diverse functions, SIRT7 has the greatest effects on the regulation of stress responses. For instance, SIRT7 is required for relieving endoplasmic reticulum stress and mitochondrial protein folding stress [42, 43]. In addition, SIRT7 can also modulate DNA repair to maintain genome integrity [37, 44–46], and sensitize cancer cells to chemotherapeutic drugs through decreasing Akt activity [47]. Intriguingly, previous studies revealed that glucose deprivation or AICAR (an AMPK activator) facilitated AMPK-triggered SIRT7 (T153) phosphorylation, leading to release of SIRT7 from the nucleoli into nucleoplasm and silencing of rDNA transcription [8, 48], as well as elevated NAD^+^ could increase SIRT7 enzyme activity during energy deficit [5, 6]. We found that SIRT7 protein levels remained unchanged during glucose deprivation. This study thus delineates an additional role for nucleoplasmic activated SIRT7 in the regulation of glucose deprivation-triggered cell-cycle arrest.

Based on activated p53 is essential for arresting cell growth during glucose limitation [13, 15], many factors (e.g., PGC-1α and Atg7) can determine cell-fate decisions by modulating p53 transactivation upon glucose starvation [23, 49]. Of note, although previous study demonstrated that SIRT7 downregulated p53’s activation by directly deacetylating p53 in cardiomyocytes [50], our data (Fig. S2D) and other studies failed to detect that SIRT7 deacetylates p53 in vitro or in cells [37, 38], which raises the question as to how SIRT7 affects p53 activity. The present study is the first to emphasize the effects of SIRT7 on p53 stability but not p53 transcriptional levels. p53 turnover is predominantly determined by MDM2-mediated ubiquitination [16, 17]. MDM2 acetylation by CBP or phosphorylation by ATM can influence p53 stabilization and activation [51, 52]. In addition, after treating cells with the radiomimetic drug neocarzinostatin, the MDM2 half-life quickly drops, resulting in p53 activation [33, 53]. Notably, our data suggested that glucose deprivation caused a marked degradation of MDM2, we consider that which is achieved through activated and phosphorylated SIRT7 in nucleoplasm [8, 48]. The interaction between SIRT7 and MDM2 remains unaltered upon glucose starvation and SIRT7 fails to deacetylate MDM2 (Fig. S3G, H), suggesting SIRT7 acts indirectly on MDM2 degradation. Indeed, we found that SIRT7-mediated MDM2 degradation was directly dependent on PCAF.

PCAF has both HAT and ubiquitination activity [4, 33], and its HAT activity is critical for maximal degradation of MDM2 [33], but PCAF does not acetylate MDM2 in vitro [51]. Posttranslational modifications are effective approaches for regulating PCAF HAT activity or cellular translocation [30–32]. However, little is known regarding the factors that regulate its E3 ligase activity. Our data reveal that SIRT7 positively modulates PCAF’s E3 ligase activity against MDM2 by directly deacetylating PCAF at K720, but still need to further explore whether PCAF K720 deacetylation has effects on its HAT activity or nuclear/cytoplasmic shuttling. Interestingly, PCAF acetylates p53 at K320 to activate p53’s DNA binding ability upon DNA damage [54]. Therefore, our study adds further evidence that PCAF is a critical regulator of p53 activity by connecting SIRT7-mediated deacetylation. Notably, our primary results showed that PCAF was also modified by phosphorylation after glucose deprivation or treating with AICAR (Fig. S5C). It is tempting to further study the role of PCAF phosphorylation in energy metabolism.

Cancer cells typically exhibit a higher rate of aerobic glycolysis to derive a significant growth advantage over normal cells —the “Warburg effect”, which is a master factor that facilitates cancer-cell resistance to therapies [55]. Thus caloric restriction is a promising approach for cancer therapy [56, 57]. 2-deoxy-D-glucose, a glycolytic inhibitor, has been considered as a potential anticancer agent [58]. SIRT7 seems to have a dual role in cancer cells by targeting substrates (e.g., H3K18ac, ATM, and SMAD4). In this study, our data add a supportive evidence for the antineoplastic function of SIRT7 [59, 60]. Namely, upon glucose deprivation, phosphorylated and activated SIRT7 is released from nucleoli into the nucleoplasm and then deacetylates PCAF, which serves as a major mechanism to control MDM2 degradation and p53 activation, resulting in cell proliferation arrest and cell death. Our study thus may provide open avenues to develop more effective tumor-starving cancer therapeutics based on the SIRT7–PCAF–p53 axis.

## Materials and methods

### Cell culture, antibodies, and reagents

p53^+/+^ HCT116 and p53^−/−^ HCT116 (human colon cancer) and U2OS (human osteosarcoma cancer) cell lines were cultured in DMEM (HyClone SH30243.01) supplemented with 10% (vol/vol) fetal bovine serum (FSP500) and 1% (vol/vol) penicillin/streptomycin (HyClone CC004) and maintained in a humidified 37 °C incubator with a 5% (vol/vol) CO_2_ atmosphere. HC116 and U2OS cell lines both acquired from ATCC. For glucose starvation, cells were cultured in DMEM no glucose medium (Gibco 11966-025). The antibodies used in this study included: anti-p21 (Cell Signaling 2947S), anti-SIRT7 (Cell Signaling 5360S), anti-acetyl-lysine (Cell Signaling 9441S), anti-p53 (DO-1 sc-126), anti-β-actin (Santa Cruz 3700S), anti-PCAF (Bethyl and Cell Signaling 3378S), anti-MDM2 (ab16895 and Cell Signaling 86934), anti-FLAG (Sigma F1804), and anti-MYC (MBL M047-3). MG132 was purchased from Sigma (M7449) and CHX was purchased from Cell Signaling (2112S).

### Plasmids and RNAi

SIRT7 and PCAF cDNAs were cloned into p3×FLAG-CMV-10, pET-28a, and pGEX-4T1 plasmids. All indicated mutations were generated using a site-directed mutagenesis kit (Vazyme). All expression constructs were verified by DNA sequencing (Shanghai Sangon Biotech Company). siRNAs (Shanghai GenePharma Company) were used to silence target genes by transient transfection with lipofectamine 2000 (Invitrogen) according to the manufacturer’s instructions. The RNAi oligonucleotide sequences were as follows:

NC siRNA: 5′-UUCUCCGAACGUGUCACGU-3′;

SIRT7 siRNA 1#: 5′-TAGCCATTTGTCCTTGAGGAA-3′;

SIRT7 siRNA 2#: 5′-GAACGGAACTCGGGTTATT-3′.

### Establishment of stable cell lines

#### shRNA knockdown

Stable SIRT7 knockdown (shSIRT7) cells were generated using shRNA constructs with the SIRT7 siRNA sequence cloned into the pGLV3/H1/GFP/Puro vector (GenePharma).

#### CRISPR-Cas9 gene editing

PCAF KO HCT116 cell line was generated as previously described via Lipofectamine 2000 transfection of small guide RNAs cloned into the pCRISPR-LvSG03 vector (GeneCopoeia) [61]. A detailed protocol has been previously reported [62]. SIRT7 KO HCT116 cell line was generated as previously described via Lipofectamine 2000-mediated transfection of small guide RNA constructs (sequence, CGAGAGCGCGGACCTGGTAA; cloned into a p×459/Puro vector) [46, 62].

### Sodium dodecyl sulfate-polyacrylamide gel electrophoresis (SDS-PAGE) and immunoblotting

Protein quantification, SDS-PAGE, and immunoblotting were used to evaluate protein levels as previously described [63]. Equal amounts of proteins were sized-fractionated by 7.5–15% (wt/vol) SDS-PAGE gels.

### Immunoprecipitation assay

Cells were harvested and then lysed in lysis buffer [1% Nonidet P-40, 20 mM Tris-HCl (pH 8.0), 137 mM NaCl, 10% glycerol, 2 mM EDTA, 1% complete protease inhibitor cocktail (Roche)] on ice for 30 min. After centrifugation at 4 °C 12,000 rpm (13,523 g) for 15 min, the supernatant was collected and incubated with the indicated antibodies (1–4 μg) at 4 °C with rotation for 8–12 h. Protein G/A-agarose beads (GE Healthcare) were then added to the sample, and incubated for a further 3 h at 4 °C. The beads were washed four times with lysis buffer and then eluted with 2 × SDS loading buffer by boiling at 100 °C for 5 min. The supernatant was analyzed by SDS-PAGE and immunoblotting.

### Real-time PCR assay

Total RNA was extracted from tumor cells by TRIzol reagent (Invitrogen) and cDNA was synthesized using ReverTra Ace qPCR RT Master Mix (TOYOBO). Quantitative real-time PCR was performed using SYBR Green Realtime PCR Master Mix (TOYOBO). The primer sequences used for *p21* and *p53* amplification were as follows:

*p21* forward, 5′-TGTCCGTCAGAACCCATGC-3′,

*p21* reverse, 5′-AAAGTCGAAGTTCCATCGCTC-3′;

*p53* forward, 5′-CAGCACATGACGGAGGTTGT-3′,

*p53* reverse, 5′-TCATCCAAATACTCCACACGC-3′.

### GST pull-down assay

GST or GST-fusion proteins were expressed in *Escherichia coli*, induced with IPTG (Sigma) at 16 or 28 °C for 8–12 h, and then pulled down with glutathione-Sepharose 4B beads (GE Healthcare). The immunoprecipitates were washed four times with buffer [20 mM Tris-HCl (pH 7.4), 0.1 mM EDTA, and 100 Mm NaCl] and then incubated with cell extracts or 6 × His-tagged fusion proteins (from *E. coli*) at 4 °C rotating overnight. After four washes with TEN buffer [0.5% Nonidet P-40, 20 mM Tris (pH 7.4), 0.1 mM EDTA, and 300 mM NaCl]. The beads were boiled in 2 × SDS loading buffer and analyzed by SDS-PAGE and immunoblotting.

### In vitro deacetylation assay

FLAG-PCAF or FLAG-SIRT7 was transfected into HCT116 cells and immunopurified by M2 beads (Sigma). For FLAG-PCAF, the beads were washed four times with lysis buffer while FLAG-SIRT7 was eluted by 3× FLAG peptide (0.125 mg/mL). For in vitro deacetylation, beads-bound FLAG-PCAF was incubated with the eluted FLAG-SIRT7 proteins in deacetylation buffer (10 mM Tris-HCl pH 8.0, 4 mM MgCl_2_, 0.2 mM DTT, 10% glycerol and 2 mM NAD^+^) at 30 °C for 1 h. The acetylated PCAF was detected by SDS-PAGE and immunoblotting.

### Identification of PCAF deacetylation site by mass spectrometry

To identify in vivo deacetylation sites of PCAF, FLAG-PCAF plasmids were transfected into SIRT7 (WT) or SIRT7 (KO) HCT116 cells. The cell lysates were co-immunoprecipitated with M2 beads after 48 h. The immunoprecipitated FLAG-PCAF was subjected to SDS-PAGE. The gels were stained with Coomassie brilliant blue (Imperial Protein Stain, Thermo), and the bands corresponding to PCAF were isolated and analyzed by LC-MS/MS.

### Flow cytometry

Cell-cycle analysis by flow cytometry was carried out, as described previously [64]. All FACS data were analyzed using ModFit software.

### Quantification and statistical analysis

Statistical analysis was performed essentially by one-way analysis or Student’s *t* test using GraphPad Prism. All experiments were performed at least three times. Sample size, *n*, for each experiment was given in the figure legends. Values represent mean ± SD. Value differences were considered significant when *p < 0.05 (not significant *p* > 0.05, **p < 0.01, ***p < 0.001).

## Supplementary information

supplementary Figure legends

supplementary Figure 1

supplementary Figure 2

supplementary Figure 3

supplementary Figure 4

supplementary Figure 5

supplementary Figure 6
