# Correction: SIRT7 activates p53 by enhancing PCAF-mediated MDM2 degradation to arrest the cell cycle

**DOI:** 10.1038/s41388-026-03881-y

**Published:** 2026-07-01

**Authors:** Ya-Fei Lu, Xiao-Peng Xu, Xiao-Peng Lu, Qian Zhu, Ge Liu, Yan-Tao Bao, He Wen, Ying-Lu Li, Wei Gu, Wei-Guo Zhu

**Affiliations:** 1 Guangdong Key Laboratory of Genome Instability and Human Disease, Shenzhen University International Cancer Center, Department of Biochemistry and Molecular Biology, Shenzhen University School of Medicine, Shenzhen, 518055 China; 2 Institute for Cancer Genetics, College of Physicians and Surgeons, Columbia University, New York, NY 10032 USA; 3 Key Laboratory of Carcinogenesis and Translational Research (Ministry of Education), Beijing Key Laboratory of Protein Posttranslational Modifications and Cell Function, Department of Biochemistry and Molecular Biology, School of Basic Medical Sciences, Peking University Health Science Center, Beijing, 100191 China; 4 Peking University—Tsinghua University Center for Life Sciences, Beijing, 100871 China

**Keywords:** Cancer therapeutic resistance, Acetylation

Correction to: *Oncogene* 10.1038/s41388-020-1305-5, published online 13 May 2020

Following the publication of this article, an unintentional duplication of the β‑actin loading control image used in Figures 1H and 2F was noted. The authors have located the original, uncropped, unedited full‑membrane scan (including molecular weight markers) for the correct β‑actin loading control for Figure 2F and a corrected version of the Figure is provided below:

The authors apologise for this unintentional error and thank the journal for the opportunity to correct the record. This correction does not affect the conclusions of the paper.

**Incorrect Figure 2F** 
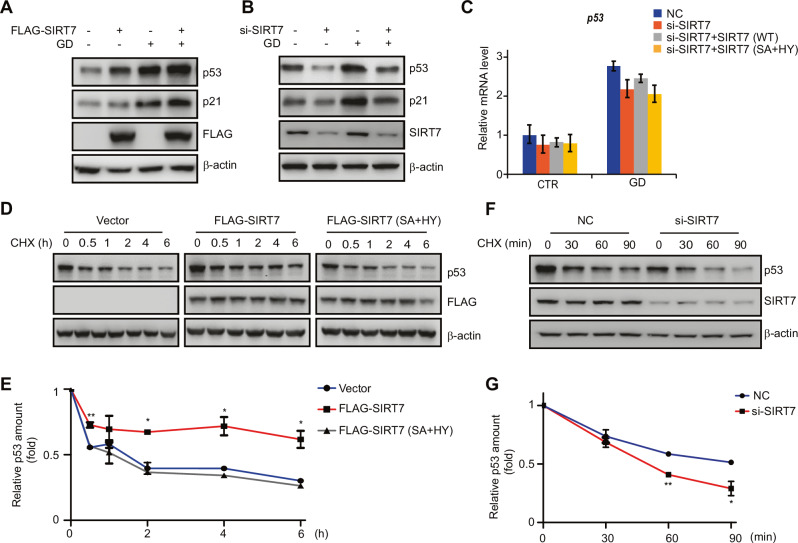


**Correct figure 2** 
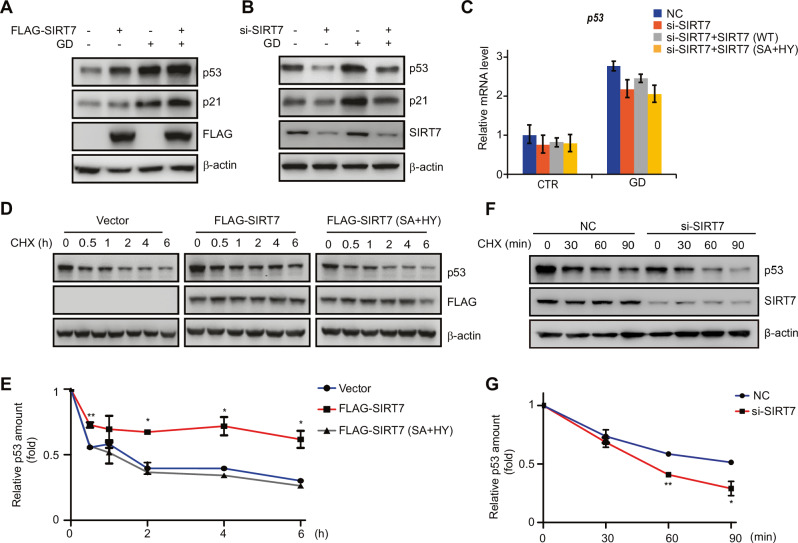


The original article has been corrected.

