# Supplementary material for: SIRT7 activates p53 by enhancing PCAF-mediated MDM2 degradation to arrest the cell cycle
Source: Oncogene. 2020 May 13;39(24):4650–65. doi: 10.1038/s41388-020-1305-5 (PMC7286819; doi:10.1038/s41388-020-1305-5)
Supplement: Supplementary file 1 — supplementary Figure legends [file 41388_2020_1305_MOESM1_ESM.docx]

Supplementary Information for

**SIRT7 activates p53 by enhancing PCAF-mediated MDM2 degradation to arrest the cell cycle**

**Ya-Fei Lu^1†^, Xiao-Peng Xu^1†^, Xiao-Peng Lu^1^, Qian Zhu^1^, Ge Liu^1^, Yan-Tao Bao^1^, He Wen^1^, Ying-Lu Li^2*^, Wei Gu^3^, Wei-Guo Zhu^1,4,5*^**

Correspondence: Wei-Guo Zhu [zhuweiguo@szu.edu.cn](mailto:zhuweiguo@szu.edu.cn)

or Ying-Lu Li [yl4187@cumc.columbia.edu](mailto:yl4187@cumc.columbia.edu)

**This file includes:**

Supplementary Figures

Fig. S1 to Fig. S6

**Fig. S1** SIRT7 is required for p53-dependent cell cycle arrest upon glucose deprivation. **A** U2OS cells were transfected with the indicated siRNAs, and then subjected or not to glucose deprivation (GD) for the indicated for the indicated times and whole cell lysates were analyzed by immunoblotting with the indicated antibodies. **B** A549 and H1299 cells were subjected to GD or not for the indicated times and whole cell lysates were analyzed by immunoblotting with the indicated antibodies. **C** p53^+/+^ and p53^-/-^ HCT116 cells were subjected to GD or not for 24 h. The whole cell lysates were analyzed by immunoblotting. β-actin was used as a loading control throughout. **D** p53^+/+^ and p53^-/-^ HCT116 cells were subjected to GD or not (CTR) for 24 h. Relative *p21* expression was determined by real-time PCR. The data represent the means ± SD (n = 3 experiments), unpaired two-tailed *t* Student, ***p<0.001, compared to the indicated group.

**Fig. S2**  SIRT7 regulates p53 activity**. A** U2OS cells were transfected with SIRT7 siRNA and subjected or not to glucose starvation (GD) for 12 h. Whole cell lysates were analyzed by immunoblotting. **B** and **C** U2OS cells were transfected with the indicated plasmids or siRNAs, then treated with cycloheximide (CHX) (30 μg/mL) for the indicated times. Whole cell lysates were analyzed by immunoblotting. Immunoblots in **B** and **C** were scanned and normalized to β-actin to quantitation of p53 protein levels. **D** SIRT7 does not deacetylate p53. HCT116 cells were transfected with the indicated siRNAs and treated with MG132 or not. Whole cell lysates were analyzed by immunoblotting.

**Fig. S3** SIRT7 promotes MDM2 degradation. **A** U2OS cells were transfected with the indicated siRNAs and then treated with cycloheximide (CHX) (30 μg/mL) for the indicated times. Whole cell lysates were analyzed by immunoblotting. **B** and **C** HCT116 cells were transfected with the indicated plasmids and siRNAs. Whole cell lysates were analyzed by immunoblotting. **D** U2OS cells were transfected with the indicated plasmids and treated with 10 μM MG132 for 8 h. The whole cell lysates were subjected to immunoprecipitation with anti-MDM2 antibodies and analyzed by immunoblotting. **E** HCT116 cells were transfected with FLAG-SIRT7 or an empty vector and treated with 10 μM MG132 for 8 h. The whole cell lysates were subjected to immunoprecipitation with anti-MDM2 antibodies and analyzed by immunoblotting. **F** Stable SIRT7 knockdown (shSIRT7) or control (shCtr) U2OS cells were subjected or not to glucose deprivation (GD) and treated with 10 μM MG132 for 12 h. The whole cell lysates were analyzed by immunoblotting. **G** HCT116 cells were subjected to 10 μM MG132 treated with or without glucose deprivation (GD) for 8 h. The whole cell extracts were immunoprecipitated with IgG or anti-MDM2 antibodies and then analyzed by immunoblotting. **H** HCT116 cells were transfected with FLAG-SIRT7 or an empty vector and treated with 10 μM MG132 for 8 h. The whole cell lysates were subjected to immunoprecipitation with anti-MDM2 antibodies and analyzed by immunoblotting.

**Fig. S4.** SIRT7 interacts with PCAF. **A** PCAF (WT) and PCAF (KO) cells were transfected with FLAG-SIRT7 or an empty vector and then treated with CHX for the indicated times. Cells were harvested and analyzed by immunoblotting. Immunoblots in left panel were scanned and normalized to β-actin to quantitate MDM2 protein levels (right panel). **B** and **C** U2OS cells lysates were immunoprecipitated with an anti-IgG, anti-SIRT7 or anti-PCAF antibody and analyzed by immunoblotting. (**D**) U2OS cells were transfected with FLAG-PCAF and subjected to glucose deprivation (GD) for 12 h. Cell lysates were immunoprecipitated with FLAG-conjugated M2 beads and then analyzed by immunoblotting.

**Fig. S5** SIRT7 deacetylates PCAF at lysine 720. **A** U2OS cells were transfected with the indicate plasmids. The whole cell lysates were immunoprecipitated with an anti-PCAF antibody and analyzed by immunoblotting. **B** U2OS cells were transfected with FLAG-PCAF and subjected to glucose deprivation (GD) for 0 h or 12 h, or re-cultured in replete growth medium for 12 h after starvation. Cell extracts were immunoprecipitated with FLAG-conjugated M2 beads before immunoblotting. **C** HCT116 cells were transfected with FLAG-PCAF and then treated with AICAR or GD. Cell extracts were immunoprecipitated with FLAG-conjugated M2 beads before immunoblotting. **D** FLAG-PCAF plasmids were transfected into SIRT7 (WT) or SIRT7 knockout (KO) HCT116 cells. The whole cell lysates were immunoprecipitated with FLAG-conjugated M2 beads. The immunoprecipitated FLAG-PCAF was subjected to SDS-PAGE. The gels were stained with Coomassie brilliant blue. The bands corresponding to PCAF were analyzed by LC-MS/MS. **E** Alignment of homologous PCAF sequences of other species around the amino acid residues adjacent to human PCAF-K720.

**Fig. S6** SIRT7 augments PCAF binding to MDM2. **A** and **B** SIRT7 does not affect PCAF protein levels. HCT116 cells were transfected with the indicated plasmids and siRNAs. The whole cell lysates were analyzed by immunoblotting. **C** Stable SIRT7 knockdown (shSIRT7) or control (shCtr) U2OS cells lysates were subjected to immunoprecipitation with an anti-MDM2 antibody before immunoblotting. **D** U2OS cells were exposed to glucose deprivation (GD) or not for 8 h. The cell lysates were subjected to immunoprecipitation with an anti-PCAF antibody before immunoblotting. **E** U2OS cells were transfected with the indicated plasmids. The cell lysates were subjected to immunoprecipitation with FLAG-conjugated M2 beads before immunoblotting.
