# Supplementary figures and images for: SIRT7 activates p53 by enhancing PCAF-mediated MDM2 degradation to arrest the cell cycle
Source: Oncogene. 2020 May 13;39(24):4650–65. doi: 10.1038/s41388-020-1305-5 (PMC7286819; doi:10.1038/s41388-020-1305-5)

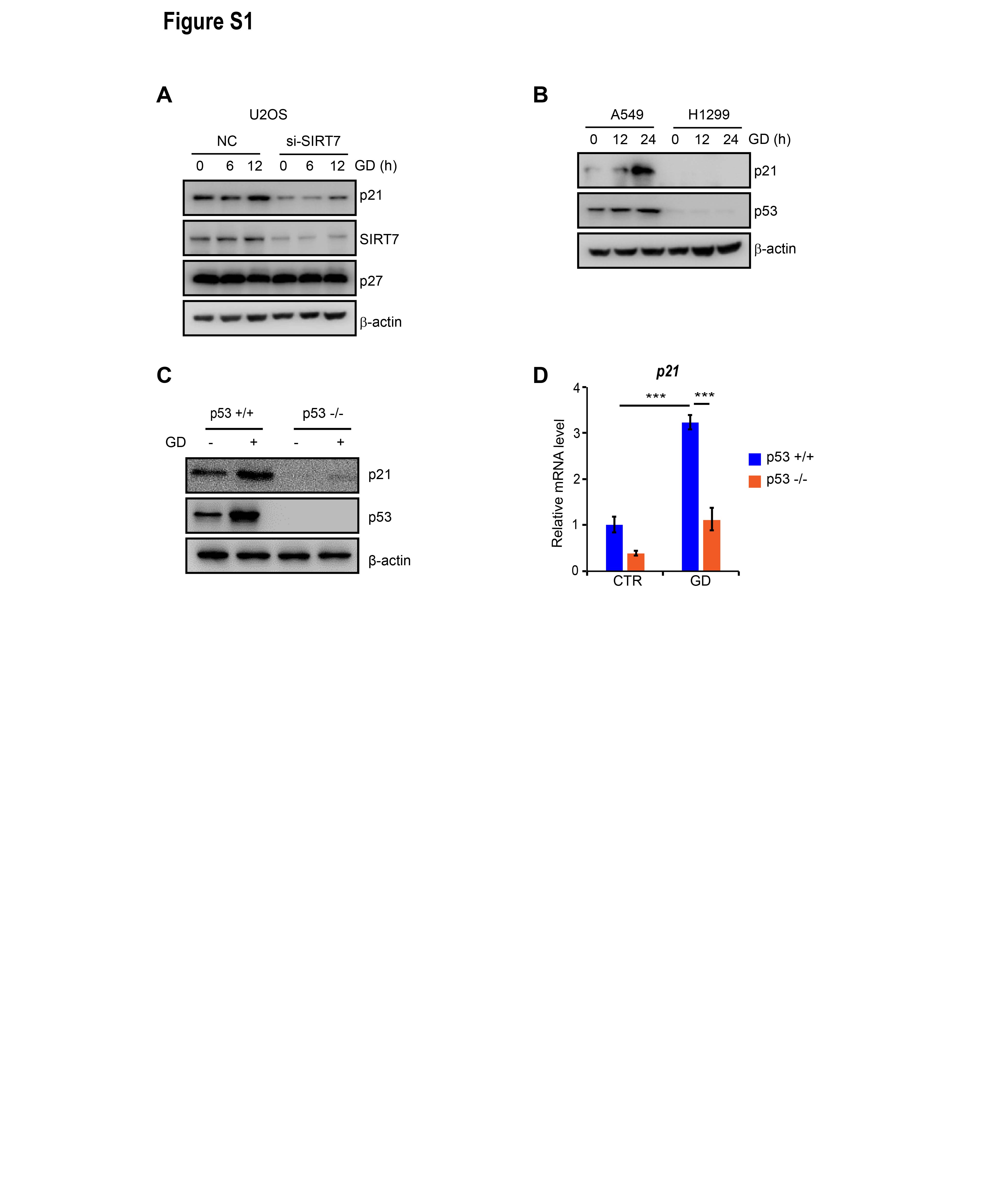

Supplement: Supplementary file 2 — supplementary Figure 1 [file 41388_2020_1305_MOESM2_ESM.jpg]

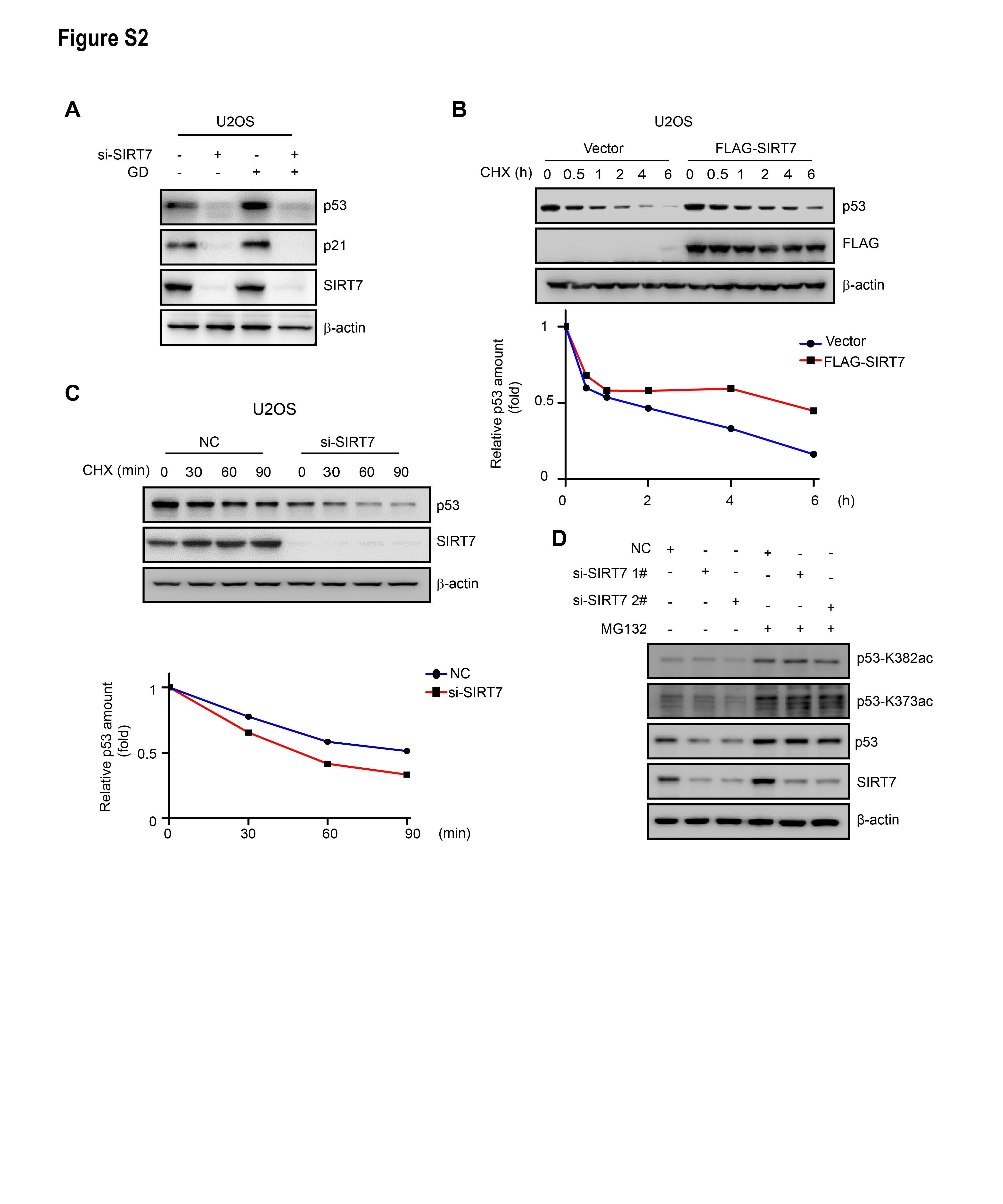

Supplement: Supplementary file 3 — supplementary Figure 2 [file 41388_2020_1305_MOESM3_ESM.jpg]

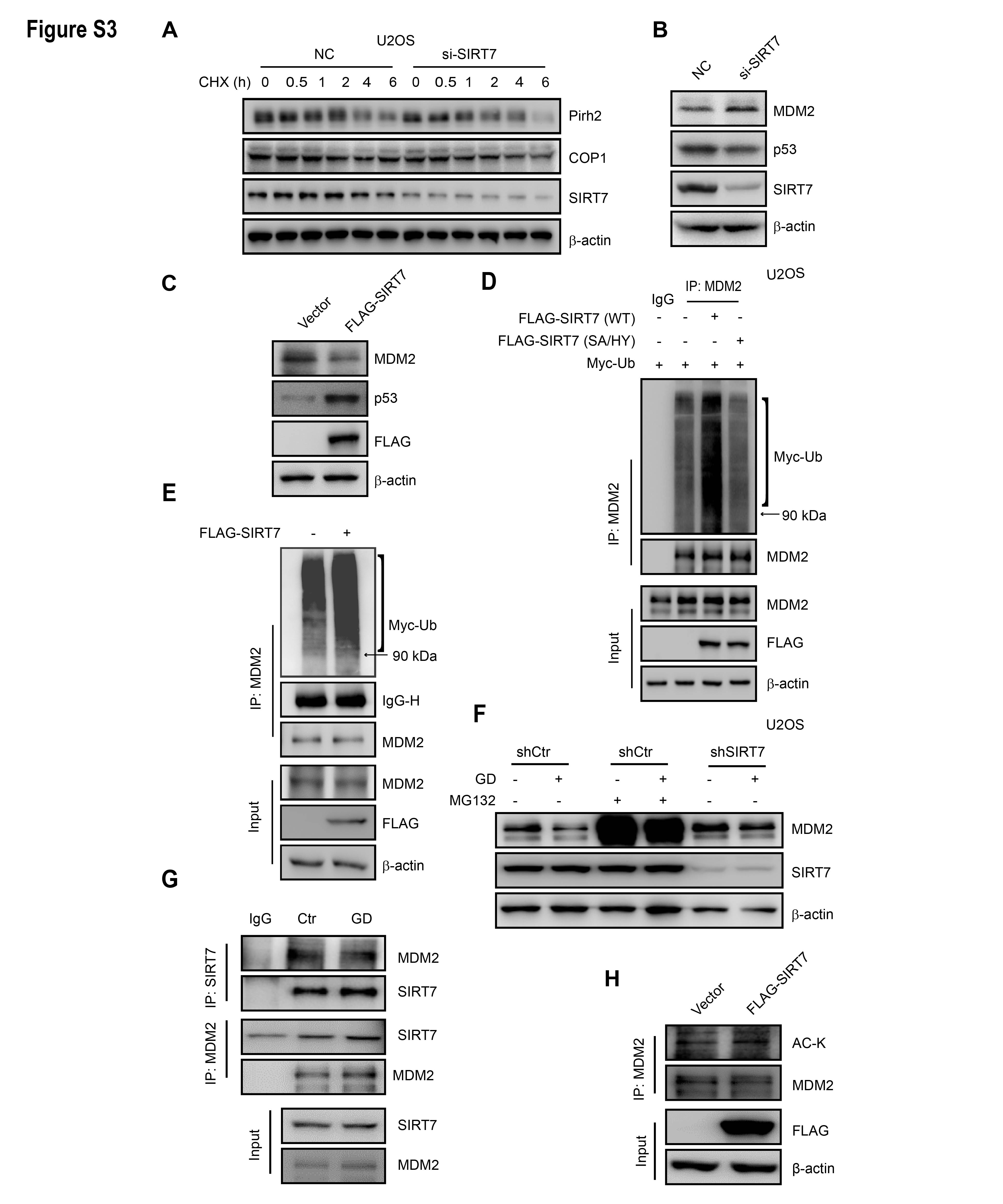

Supplement: Supplementary file 4 — supplementary Figure 3 [file 41388_2020_1305_MOESM4_ESM.jpg]

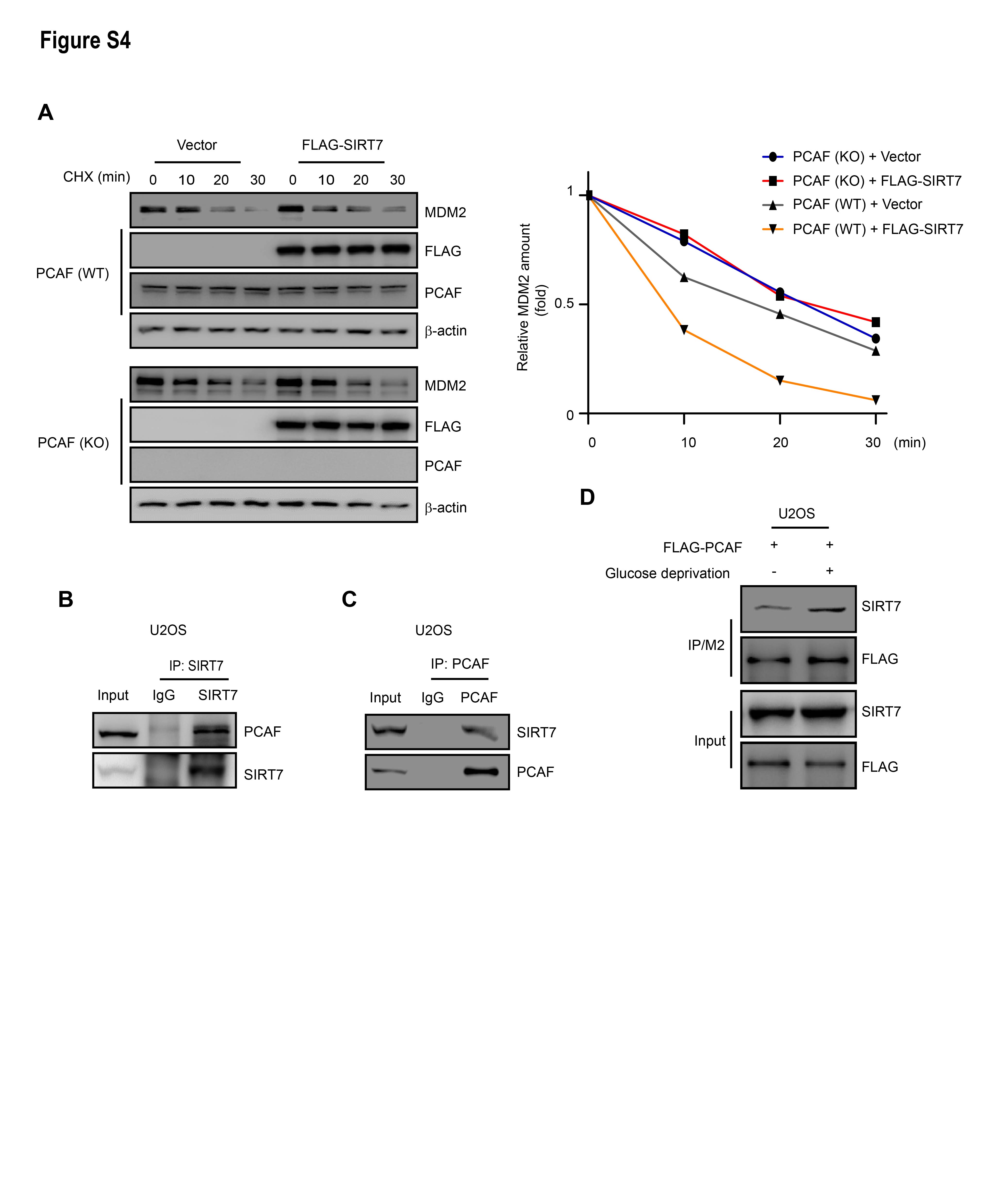

Supplement: Supplementary file 5 — supplementary Figure 4 [file 41388_2020_1305_MOESM5_ESM.jpg]

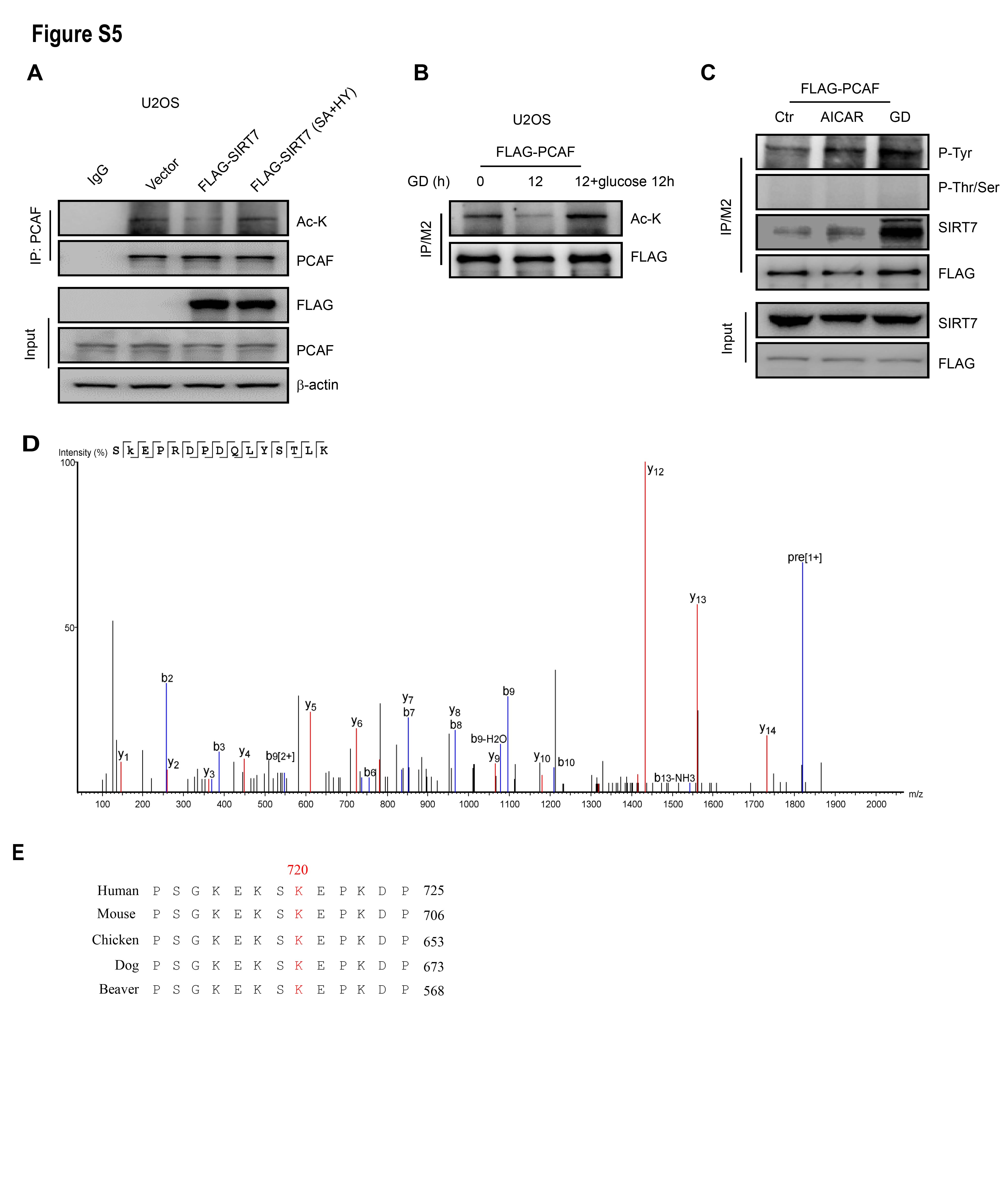

Supplement: Supplementary file 6 — supplementary Figure 5 [file 41388_2020_1305_MOESM6_ESM.jpg]

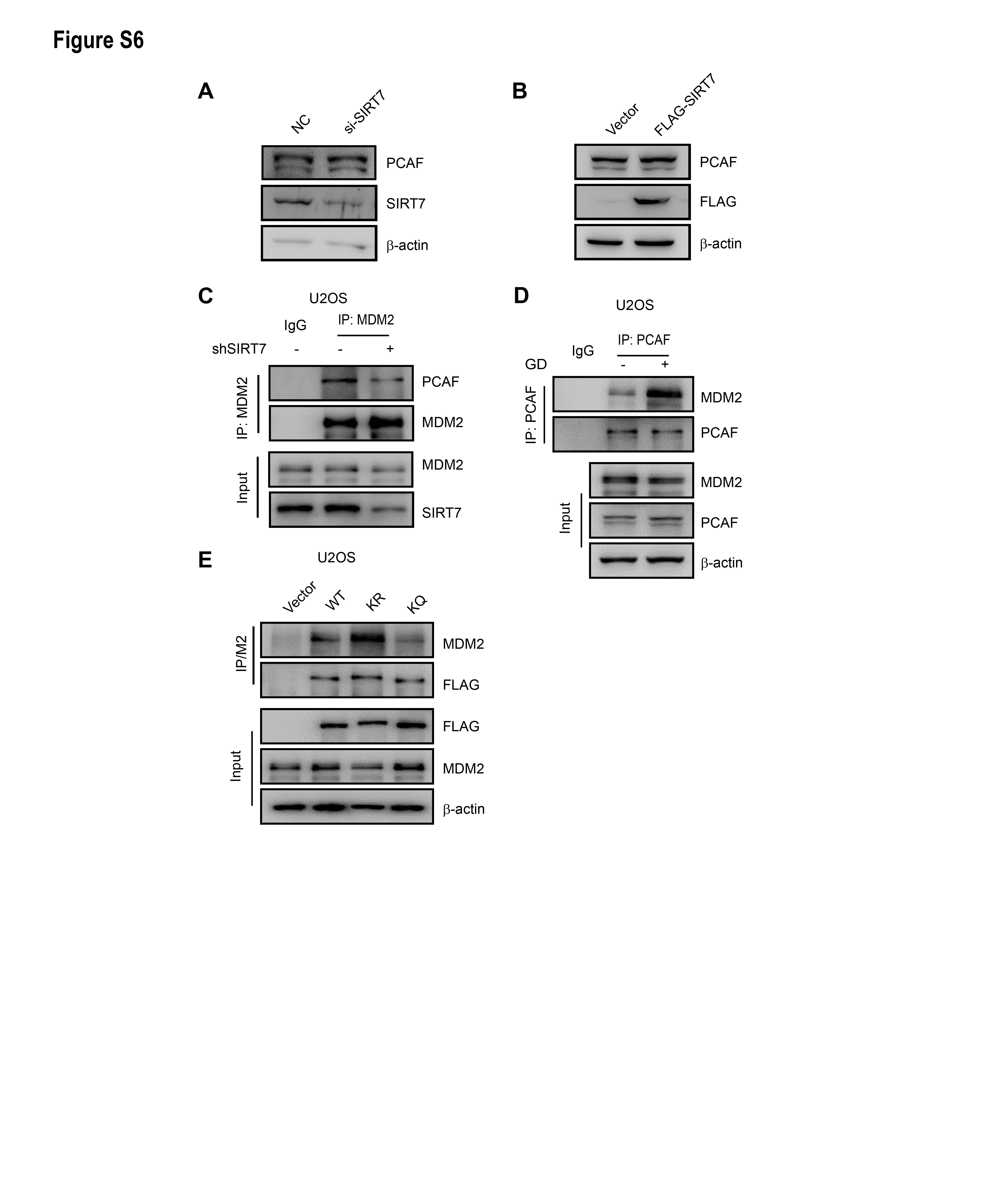

Supplement: Supplementary file 7 — supplementary Figure 6 [file 41388_2020_1305_MOESM7_ESM.jpg]
